# Opportunistic Pathogen *Porphyromonas gingivalis* Modulates Danger Signal ATP-Mediated Antibacterial NOX2 Pathways in Primary Epithelial Cells

**DOI:** 10.3389/fcimb.2017.00291

**Published:** 2017-07-04

**Authors:** JoAnn S. Roberts, Kalina R. Atanasova, Jungnam Lee, Gill Diamond, Jeff Deguzman, Chul Hee Choi, Özlem Yilmaz

**Affiliations:** ^1^Department of Oral Health Sciences, Medical University of South CarolinaCharleston, SC, United States; ^2^Department of Periodontology, University of FloridaGainesville, FL, United States; ^3^Department of Oral Biology, University of FloridaGainesville, FL, United States; ^4^Department of Microbiology and Medical Science, School of Medicine, Chungnam National UniversityDaejeon, South Korea; ^5^Department of Microbiology and Immunology, Medical University of South CarolinaCharleston, SC, United States

**Keywords:** opportunistic oral bacteria, persistence, ROS, NADPH oxidase, myeloperoxidase, glutathione, danger signal ATP

## Abstract

*Porphyromonas gingivalis*, a major opportunistic pathogen in the etiology of chronic periodontitis, successfully survives in human gingival epithelial cells (GECs). *P. gingivalis* abrogates the effects of a host danger molecule, extracellular ATP (eATP)/P2X_7_ signaling, such as the generation of reactive oxygen species (ROS) via the mitochondria and NADPH oxidases (NOX) from primary GECs. However, antimicrobial functions of ROS production are thoroughly investigated in myeloid-lineage immune cells and have not been well-understood in epithelial cells. Therefore, this study characterizes antibacterial NOX2 generated ROS and host downstream effects in *P. gingivalis* infected human primary GECs. We examined the expression of NOX isoforms in the GECs and demonstrate eATP stimulation increased the mRNA expression of NOX2 (*p* < 0.05). Specific peptide inhibition of NOX2 significantly reduced eATP-mediated ROS as detected by DCFDA probe. The results also showed *P. gingivalis* infection can temporally modulate NOX2 pathway by reorganizing the localization and activation of cytosolic molecules (p47phox, p67phox, and Rac1) during 24 h of infection. Investigation into downstream biocidal factors of NOX2 revealed an eATP-induced increase in hypochlorous acid (HOCl) in GECs detected by R19-S fluorescent probe, which is significantly reduced by a myeloperoxidase (MPO) inhibitor. MPO activity of the host cells was assayed and found to be positively affected by eATP treatment and/or infection. However, *P. gingivalis* significantly reduced the MPO product, bactericidal HOCl, in early times of infection upon eATP stimulation. Analysis of the intracellular levels of a major host-antioxidant, glutathione during early infection revealed a substantial decrease (*p* < 0.05) in reduced glutathione indicative of scavenging of HOCl by *P. gingivalis* infection and eATP treatment. Examination of the mRNA expression of key enzymes in the glutathione synthesis pathway displayed a marked increase (*p* < 0.05) in glutamate cysteine ligase (GCL) subunits GCLc and GCLm, glutathione synthetase, and glutathione reductase during the infection. These suggest *P. gingivalis* modulates the danger signal eATP-induced NOX2 signaling and also induces host glutathione synthesis to likely avoid HOCl mediated clearance. Thus, we characterize for the first time in epithelial cells, an eATP/NOX2-ROS-antibacterial pathway and demonstrate *P. gingivalis* can circumvent this important antimicrobial defense system potentially for successful persistence in human epithelial tissues.

## Introduction

Epithelial cells, as the first line of defense in the oral cavity and other mucosal regions in the body, are continuing to unfold as a critical cell type central to the host immune system for appropriate recognition and responses against invading microbes and pathogens (Sandros et al., 2000; Dale, 2002, 2003; Sugawara et al., 2002; Artis, 2008). Part of this well-orchestrated host innate response to stress or infection stimuli includes the release of small “danger signaling molecules” such as ATP which functions as a sensor against colonizing pathogens through activation of the purinergic receptor P2X_7_ (Gordon, 1986; Schwiebert and Zsembery, 2003; Trautmann, 2009; Yilmaz et al., 2010; Almeida-da-Silva et al., 2016). Activation of the P2X_7_ receptor mediates multiple responses including induction of apoptosis, increased reactive oxygen species (ROS), and microbial elimination (Gordon, 1986; Schwiebert and Zsembery, 2003; Yilmaz, 2008; Miller et al., 2011; Spooner and Yilmaz, 2011; Hung et al., 2013). ROS generated specifically in response to extracellular ATP (eATP) signaling has been identified from two main sources, the mitochondria and NADPH oxidases (NOX) which can work synergistically to promote ROS generation during microbial infection (Dikalov, 2011; Fang, 2011; Spooner and Yilmaz, 2011; Roberts and Yilmaz, 2015). ROS produced in response to invading pathogens have been mostly studied as a toxic agent for microbial elimination which may promote host bacterial elimination machinery such as autophagy (Hampton et al., 1998; Lambeth, 2004; Fang, 2011; Paiva and Bozza, 2014; Roberts and Yilmaz, 2015). The oxidative bacterial killing mechanisms in neutrophils have been clearly described by decades of research highlighting the role of NOXs during the phagocytosis of an invading pathogen (Hampton et al., 1998; Cross and Segal, 2004; Lambeth, 2004). Therefore, the accepted paradigm for clearance of bacteria has been established in neutrophils through the respiratory burst which includes the generation of superoxide from the NOX2 isoform which is rapidly converted into H_2_O_2_ by superoxide dismutase (Cross and Segal, 2004; Nauseef, 2007, 2014). The H_2_O_2_ is then converted to hypochlorous acid (HOCl) by myeloperoxidase enzyme (MPO) to neutralize the phagocytosed bacteria (Hampton et al., 1998; Cross and Segal, 2004; Nauseef, 2007, 2014).

**Figure 1:**
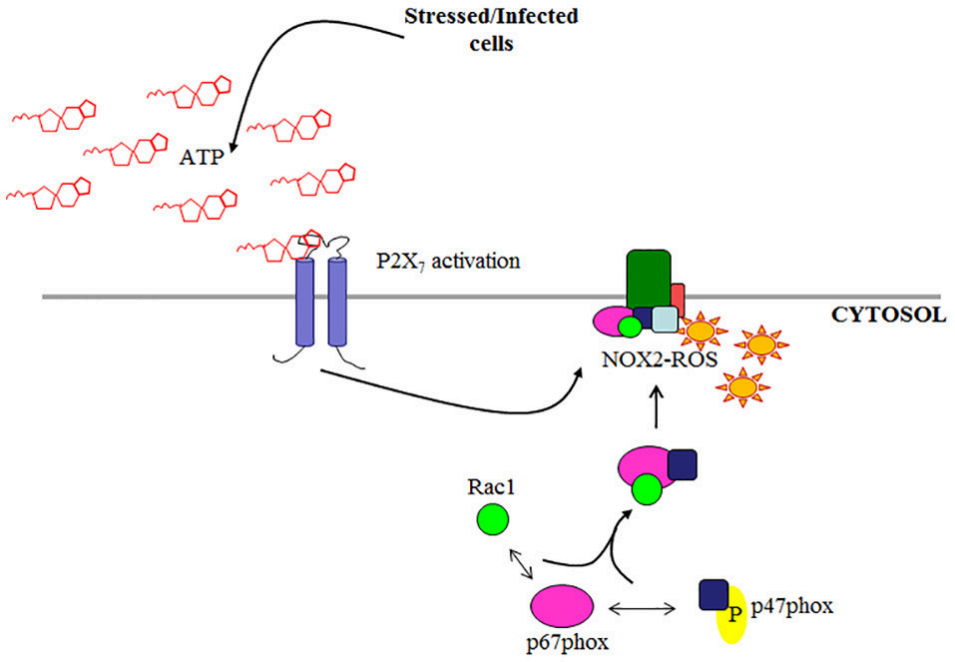
Schematic of NOX2 complex assembly. NOX2 is activated by cytosolic subunits which are recruited to the membrane subunit gp91phox under stimulated conditions (e.g., eATP/P2X_7_ signaling).

Although, there are seven known isoforms in the NADPH oxidase family (NOX1-5; DUOX1; DUOX2; Cross and Segal, 2004; Lambeth, 2004; Bedard and Krause, 2007), NOX2 is the most characterized isoform specifically in microbial elimination. In-depth studies of NOX2 in professional phagocytes have extensively described the interaction of the cytosolic regulatory subunits required for activation and the process has since recently been reviewed (Cross and Segal, 2004; Lambeth, 2004; Miyano et al., 2006; Bedard and Krause, 2007; El-Benna et al., 2009; Panday et al., 2015; p47phox, Rac1, p67phox, and p40phox). Under stimulated conditions, p47phox undergoes phosphorylation and is critical in facilitating the organization of NOX2 assembly (Hoyal et al., 2003; El-Benna et al., 2009; Meijles et al., 2014) by translocation of p67phox and p40phox to the membrane where it associates with membrane subunit gp91phox. To complete the formation of an active NOX2 complex, GTP-bound Rac1 is recruited to the membrane bound to p67phox, a required step for a functional NOX2 complex (Figure 1; Gorzalczany et al., 2000; Miyano et al., 2006; Panday et al., 2015). There is increasing evidence showing NOX2 can be expressed in other cell types other than professional phagocytes such as endothelial cells and colon epithelial cells (Kolářová et al., 2010; Regmi et al., 2014). Despite this, NOX2-ROS has not been studied in these cell types for anti-microbial functions but rather as secondary or regulatory signals (Kolářová et al., 2010; Dikalov, 2011; Fang, 2011; Paiva and Bozza, 2014; Regmi et al., 2014). However, the increasing knowledge of the role of epithelium in innate immunity against invading bacteria generates the need for characterization of potential antibacterial pathways such as NOX2 in the epithelial cells.

In periodontal disease, the opportunistic pathogen, *Porphyromonas gingivalis*, manipulates the gingival epithelial cells (GECs) to promote microbial survival and persistence in the oral mucosa (Lamont and Yilmaz, 2002; Colombo et al., 2006, 2007). *P. gingivalis*, a Gram (−) strict anaerobe and successful colonizer of the oral cavity, has been termed a keystone pathogen due to its ability to promote a host microbial environment associated with disease (Hajishengallis et al., 2012; Spooner et al., 2016). Moreover, *P. gingivalis* infection has been linked to several systemic chronic diseases such as rheumatoid arthritis, diabetes, and cancer (Atanasova and Yilmaz, 2015). Multiple immune evasion strategies are continually being defined for the microorganism in a variety of cell types (Dorn et al., 2002; Yilmaz, 2008; Hajishengallis, 2011; Choi et al., 2013; Olsen and Hajishengallis, 2016). In human GECs, *P. gingivalis* can multiply and survive intracellularly for extended periods of time and later can spread from cell-to-cell through actin formed structures to evade immune surveillance (Yilmaz et al., 2006). This gives *P. gingivalis* a unique ability to persist in the oral epi-mucosal tissues thereby being a major contributor to the progression of chronic periodontitis (Spooner et al., 2016). *P. gingivalis* achieves successful colonization in GECs by inhibiting intrinsic apoptotic pathways mediated by cytochrome *c* release and caspase 3/9 activation induced by pro-apoptotic agents (Yilmaz et al., 2004; Yao et al., 2010). Infection also abrogates eATP-induced host cell death and modulates eATP-induced cellular ROS and oxidative stress pathways (Yilmaz et al., 2008, 2010; Spooner and Yilmaz, 2011; Choi et al., 2013; Hung et al., 2013; Spooner et al., 2014; Johnson et al., 2015). Interestingly, *P. gingivalis* infection in GECs is also shown to attenuate IL-1β secretion upon secondary signal eATP stimulation which has been attributed to the NOD3/inflammasome pathway and P2X_7_ signaling (Yilmaz et al., 2010; Choi et al., 2013; Hung et al., 2013; Johnson et al., 2015). Therefore, mechanisms studied thus far reveal that *P. gingivalis* promotes subsistence in the gingival epithelium by attenuation of both pyroptotic and apoptotic pathways.

GECs are in direct contact with bacterial challenge in the oral cavity, and serve as an important model system to dissect the interactions between pathogen and innate immune response at mucosal surfaces (Sandros et al., 2000; Sugawara et al., 2002; Artis, 2008). Our previous studies showed that *P. gingivalis* can overcome initial host antibacterial responses in the human GECs by diminishing eATP/P2X_7_ receptor mediated robust and sustained ROS production through its secreted effector, nucleoside-diphosphate kinase (ndk) thereby converting these epithelial cells into a niche for intracellular survival and proliferation (Spooner and Yilmaz, 2012; Choi et al., 2013; Johnson et al., 2015; Atanasova et al., 2016). Thus, there has been increasing evidence suggesting specific crosstalk between eATP/P2X_7_ and NOX2 mediated ROS (Moore and MacKenzie, 2009; Martinon, 2010; Choi et al., 2013; Roberts and Yilmaz, 2015), which generates high interest as to the significance of these pathways in host response to *P. gingivalis*. Nevertheless, there is currently no research in epithelial cells identifying specific molecular pathways such as NOX for anti-bacterial ROS, which are also involved in eATP-mediated ROS.

In this study, using a primary GEC model, we characterize for the first time NOX2 as a key NADPH oxidase in human gingival epithelium involved in the danger signal eATP-induced ROS production resulting in significant HOCl generation via functional expression of MPO; a novel host defense response to both a danger stimulus ATP and *P. gingivalis* infection. Furthermore, we demonstrate *P. gingivalis* infection temporally modulates NOX2 activation to attenuate the eATP-mediated ROS antibacterial pathway in GECs and potentially to counteract the bactericidal effects of HOCl through the induction of host glutathione antioxidant system, which may help promote the microorganisms' colonization in the oral mucosa.

## Methods

### Bacteria and cell culture

The *P. gingivalis* ATCC strain 33277 was cultured under anaerobic conditions in Trypticase soy broth (TSB) supplemented with yeast extract (1 μg/ml), menadione (1 μg/ml), and hemin (5 μg/ml), at 37°C and harvested as described previously (Choi et al., 2013). The number of bacteria for infection was determined at mid-log phase using a Klett-Summerson photometer (Klett) and used at a multiplicity of infection (MOI) of 100 in all infection experiments. All the experiments were performed in our Biosafety Level 2 laboratory approved by the institutional biosafety committee, protocol number 530. Primary cultures of GECs were generated and cultured as described previously (Yilmaz et al., 2003, 2008). At least three different collected patient cells were used independently for the experiments and were tested for viability when treated with inhibitors or other chemical reagents in the assays performed for this study. The data represents statistical analyses of the three separate patient cells and their individual replication experiments. No human subject recruitment *per-se* was done. Adult patients were selected at random and anonymously from those presenting at the University of Florida Dental Clinics for tooth crown lengthening or impacted third molar extraction. No patient information was collected. Gingival tissue that would otherwise be discarded was collected after informed consent was obtained by all patients under the approved guidance of the University of Florida Health Science Center Institutional Review Board (IRB, human subjects assurance number FWA 00005790).

### NOX isoform expression detected by qPCR

The mRNA expression of each NOX isoform was measured by quantitative real time PCR, using specific TaqMan primers (Applied Biosystems) for each. RNA was isolated using Qiagen RNeasy Plus Kit (Qiagen) and 10 μg per sample was reverse-transcribed from untreated and 3 mM ATP (Sigma) treated primary GECs; the ATP concentration determined and used in our previous studies (Yilmaz et al., 2010; Choi et al., 2013; Hung et al., 2013; Johnson et al., 2015; Atanasova et al., 2016). cDNA were constructed using Superscript III First Strand cDNA Synthesis Kit (Invitrogen) and quantitative PCR was conducted at 95°C for 10 min, followed by 45 cycles at 95°C for 15 s, and at 60°C for 60 s. Gene expression analysis was performed using the CFX Manager Software (BioRad). The expression of reference gene GADPH was used to normalize the measured target gene. The untreated control was used to further normalize the relative quantities for the *NOX* genes.

TaqMan Primers used from Applied Biosystems.

**Table:** 

**Gene**	**Human Assay ID**
NOX1	Hs01071088_m1
CYBB/NOX2	Hs00166163_m1
NOX3	Hs01098883_m1
NOX4	Hs00418356_m1
NOX5	Hs00225846_m1
DUOX1	Hs00213694_m1
DUOX2	Hs00204187_m1
GAPDH	Hs00104187_m1

### NADP/NADPH ratio measurement

Cells were seeded in T25 flasks and treated with 3 mM ATP (Sigma) and/or infected with *P. gingivalis* over a 24 h time period. The cells (4 × 10^6^) were collected using 300 μL of extraction buffer and the NADP/NADPH levels were measured using the NADP/NADPH Assay Kit (Abcam, Cambridge, UK; ab65349). A time course was performed at 30 min intervals to determine optimal incubation time for detection at room temperature and read on a Biotek H1M monochromatic plate reader at 450 nm. The NADP and NADPH measured levels were calculated by comparison with the standard curve. Protein concentration was determined and normalized for each sample prior to analysis.

### Measurement of ROS production

Cells were incubated with 3 mM ATP in a 24-well plate or 4-well microscopic slide chamber system (Warner instruments). ATP was added to the cells for 30 min and then incubated with the peptide NOX2 inhibitor gp91ds-tat or gp91ds-tat scrambled (non-targeting) control peptide (Anaspec, Fremont, CA) at a concentration of 50 μM determined by dose response assay and the previous literature (Supplementary Figure 1). The effective delivery of this peptide into the GECs was confirmed using FAM-labeled gp91ds-tat (Anaspec, Fremont, CA) peptide (not shown). Cells were pre-labeled with the fluorescent probe 5-(and-6)-chloromethyl-2′,7′-dichlorodihydrofluorescein diacetate, acetyl ester (CM-H_2_DCFDA) (Invitrogen, Waltham, MA) which was solubilized in dimethyl sulfoxide (DMSO) (Sigma, St. Louis, MO) and then diluted to 5 μM in Hanks balanced salt solution (HBSS) containing MgCl_2_ and CaCl_2_ (Invitrogen). The cells were washed twice with HBSS, incubated with 5 μM CM-H_2_DCFDA at 37°C for 30 min in a fresh medium. The CM-H_2_DCFDA (green) fluorescence intensity was measured using a Biotek H1M monochromatic bottom fluorescence plate reader at excitation 495 nm and emission 525 nm. Results are averaged data from three individual experiments performed in duplicate and normalized to untreated cells as a control. The representative live images of the CM-H_2_DCFDA (green) fluorescence intensity were taken using epifluorescence (DM IRE2 HC inverted scope, Leica Microsystems GmbH) microscopy equipped with DIC. The single exposure images were collected sequentially in fluorescence and DIC using a Retiga 4000r CCD camera (Qimaging).

### Subcellular fractionation and western blotting of NOX2 cytosolic subunits

GECs were seeded in 6-well plates (2.5 × 10^5^) and treated with 3 mM ATP for 1 h prior to *P. gingivalis* infection for 1, 3, and 24 h. The cell lysates were collected and fractionated into cytosolic, membrane, and nuclear fractions according to the manufacturer's protocol using a Subcellular Fractionation Kit (Pierce Technologies, #87790). Samples were loaded on a 10% SDS-page gel and run at 140 V for 1 h. The separated proteins were transferred to a nitrocellulose membrane by wet transfer at 50 V for 1 h and blocked with 5% non-fat milk in 1x Tris-buffered saline and 0.1% Tween-20. The membrane was incubated overnight in 1:1,000 rabbit p67phox (Millipore), 1:1,000 mouse Rac1 (Millipore) and 1:1,000 mouse β-tubulin (Invitrogen) antibodies, washed, and incubated with secondary rabbit or mouse HRP conjugated antibodies (Invitrogen) for 1 h. Membranes were incubated with enhanced chemiluminescence (GE Healthcare Life Sciences) and exposed by autoradiography. This experiment was performed in triplicate.

### Epifluorescence microscopy

GECs were seeded at a density of 8 × 10^4^ on 17 mm glass coverslips (Warner Instruments) in four-well plates (Thermo Fisher Scientific). Cells were treated first with 3 mM ATP for 1 h and then infected with *P. gingivalis* for 3 and 24 h. The cells were fixed with 10% neutral buffered formalin, permeabilized by 0.1% Triton X-100 and blocked with 3% BSA for 45 min. The cells were stained for 1 h at room temperature with a mouse polyclonal antibody against Rac1 (1:500; Millipore) and a rabbit polyclonal antibody against *P. gingivalis* 33277 (1:1,000). The stained cells were washed and further incubated for 1 h at room temperature with Alexa Fluor 594 conjugated secondary goat anti-mouse polyclonal antibody and Alexa Fluor 488 conjugated secondary goat anti-rabbit polyclonal antibody (1:1,000; Invitrogen). The actin cytoskeleton was labeled using Alexa Fluor 350 phalloidin (1:500; Invitrogen). Coverslips with fixed cells were mounted onto Corning glass microscopy slides (Corning) using VectaShield mounting medium containing DAPI (Vector Laboratories). Images were acquired using Zeiss Axio Imager A1 epifluorescence microscope using QImaging MicroPublisher 3.3 cooled microscope camera and QCapture software.

### Immunoprecipitation of p47phox

Cells were seeded in T25 flasks at 70% confluency and treated with 3 mM ATP and *P. gingivalis* infection for 1, 3, and 24 h. ATP was added 1 h prior to infection. Cell lysates were collected in 1x RIPA buffer with protease inhibitor cocktail (Pierce Technologies) and protein levels determined by Bradford assay. The same protein quantity of lysates were incubated with 1 μg p47phox antibody (Novus Biologicals) for 1 h and further incubated with 20 μL Protein A/G bead plus (Santa Cruz) for 2 h at room temperature. The captured protein was collected in 40 μL 1x Laemmli Sample Buffer and loaded onto a 10% SDS-Page gel following the Western blot protocol described above. The blot was incubated with mouse phosphoserine antibody (1:1,000; Millipore) and mouse β-tubulin loading control (1:1,000; Invitrogen). Clean blot secondary antibody detection system designed to reduce background from immunoprecipitated samples (Pierce Technologies) at a 1:400 final concentration.

### MPO expression

MPO was visualized using immunoperoxidase staining in untreated, ATP (3 mM) or H_2_O_2_ (100 μM) treated cells; the concentrations determined and used in our previous studies (Yilmaz et al., 2010; Choi et al., 2013; Hung et al., 2013; Johnson et al., 2015; Atanasova et al., 2016). The following protocol was used for immunoperoxidase staining: Cells were fixed in 10% NBF for 30 min at RT and permeabilized for 15 min using 0.1% TritonX-100. Endogenous peroxidase activity was quenched by incubating the cells for 30 min in 0.3% hydrogen peroxide in methanol. Cells were washed and then blocked in 3% BSA in PBS-Tween and incubated for 45 min at room temperature. Cells were incubated with the primary mouse MPO antibody (R&D systems; 2.5 μg/mL) overnight at 4°C. After 30 min of 10-min washes, cells were incubated with anti-mouse HRP-labeled secondary antibody (Invitrogen; 1:100) for 1 h. After again washing, DAB-peroxidase solution was added to the cells and incubated for 10 min in the dark. Cells were then imaged using DM IRE2 HC inverted scope, Leica Microsystems at 40x magnification. ImageJ software analysis was used to measure relative intensity of the peroxidase stain and compared to untreated.

### MPO ELISA

Cell lysates of 3 mM ATP treated and *P. gingivalis*-infected or uninfected GECs were used for the quantitative measurement of MPO by ELISA (R&D systems; DY3174). Cells pre-treated with ATP and/or *P. gingivalis* were collected in 200 μL of Reagent diluent (1% BSA in PBS), disrupted by sonication three times for 5 s on ice, and clarified at max speed for 5 min. MPO-ELISA was performed following the manufacturers protocol on Corning disposable sterile ELISA plates (Corning). The optical density was read at absorbance of 450 nm using a Biotek H1M monochromatic plate reader with wavelength correction of 540 nm. A standard curve was performed at two-fold dilutions from 2,000 to 31.25 pg/mL.

### MPO activity assay (fluorometric)

GECs were seeded at 70% confluence in T25 flasks and treated with 3 mM ATP, 100 μM H_2_O_2_ or *P. gingivalis* infection. ATP was added 1 h prior to *P. gingivalis* infection. The cells were collected in 100 μL assay buffer provided by the MPO activity assay kit and sonicated 3x on ice for 5 s each, then left for 10 min on ice per the manufacturers protocol (Abcam, ab111749). The fluorescence produced was measured on a Biotek H1M monochromatic plate reader kinetically at 2-min intervals for 30 min with an excitation of 484 nm and emission of 525 nm.

### Measurement of HOCl production

GECs (8 × 10^4^) were pre-treated with 3 mM ATP, *P. gingivalis* for 2 h, 1 μM MPO inhibitor 4-Aminobenzoic hydrazide (Santa Cruz) or 50 μM gp91ds-tat/gp91ds-tat scrambled peptides (Anaspec) and then incubated for 15 min in the dark with 10 μM R19S fluorescent probe (FutureChem, South Korea). The R19S probe fluorescence was measured using the Biotek H1M monochromatic plate reader at an excitation of 515 nm and emission of 545 nm. Live fluorescent images were taken under a (DM IRE2 HC inverted scope, Leica Microsystems) microscopy equipped with DIC.

### Measurement of intracellular glutathione

The intracellular reduced glutathione was quantified using ThiolTracker Violet stain (Invitrogen) in living cells, since glutathione represents the majority of intracellular free thiols in the cell. Briefly, primary GECs were treated with or without 3 mM ATP for 1 h and/or infected with *P. gingivalis* (MOI100) in 4-well microscopic slide chamber system (LabTek) or 24-well plate. After the designated incubation period, the cells were washed and incubated in Dulbecco's PBS containing 10 μM ThiolTracker Violet for 30 min at 37°C in the dark. The cells were then read on the Biotek H1M monochromatic plate reader at excitation 405 nm and emission 525 nm. GECs seeded in 4-well chamber slides were fixed with 10% neutral buffered formalin, permeabilized by 0.1% Triton X-100 and blocked with 3% BSA for 30 min. *P. gingivalis* was immunostained using the rabbit polyclonal *P. gingivalis* antibody (1:1,000) and secondary antibody Alexa Fluor 594 (1:1,000; Invitrogen). The slides were viewed using inverted epifluorescence microscopy (DM IRE2 HC inverted scope, Leica Microsystems GmbH) and images taken using a Retiga 4000r CCD camera (Qimaging). H_2_O_2_ (100 μM) was used as a positive control for oxidative stress and the antioxidant N-acetyl cysteine (NAC) (50 mM) as an inhibitor of oxidative stress.

### SybrGreen quantitative real-time PCR

Transcription of the Glutathione ligase genes (GCLc and GCLm), Glutathione synthetase and glutathione reductase were measured by quantitative real time PCR using specific primers and the SybrGreen detection system. RNA was isolated using Qiagen RNeasy Plus Kit (Qiagen) and 1 μg per sample was reverse-transcribed from untreated, *P. gingivalis* infected, and/or 3 mM ATP treated primary GECs. cDNA was constructed using Superscript III First Strand cDNA Synthesis Kit (Invitrogen). Quantitative PCR was conducted at 95°C for 3 min and 40 cycles at 95°C for 30 s and 61°C for 30 s. Gene expression analysis was performed using the CFX Manager Software (BioRad). The expression of the reference gene GADPH was used to normalize the measured target gene and the untreated control was used to further normalize the relative quantities.

SybrGreen Primers.

**Table:** 

**Gene**	**Primers**
GCLc	F: CTGTTGCAGGAAGGCATTGAT
	R: TTCAAACAGTGTCAGTGGGTCTCT
GCLm	F: GGCACAGGTAAAACCAAATAGTAAC
	R: CAAATTGTTTAGCAAATGCAGTCA
Glutathione synthetase	F: CCCTGGCTGAGGGAGTATTG
	R: TGCACAGCATAGGCTTGCTC
Glutathione reductase	F: CACGAGTGATCCCAAGCCC
	R: GGCGGTGTACTTTTTCCCAC
GAPDH	F: GAAATCCCATCACCATCTTCCAGG
	R: GAGCCCCAGCCTTCTCCATG

### Statistical analysis

All assays were performed in at least three separate occasions in duplicate or triplicate each time. Results are expressed as mean ± SEM. Two-tailed Student's t-test was used to calculate the statistical significance of the experimental results between two conditions (significance considered at *p* < 0.05).

## Results

### NOX2 is a major ROS producer in response to ATP stimulation in primary GECs

**Figure 2:**
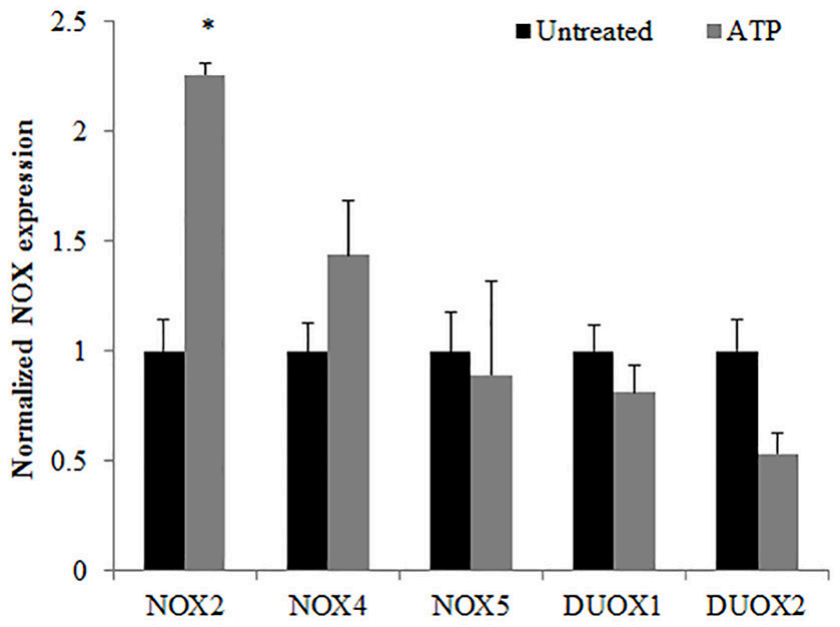
Characterization of NOX isoforms in Primary GECs with ATP stimulation. TaqMan qPCR detection of mRNA expression levels of NADPH oxidase isoforms in human primary GECS with and without ATP (3 mM) treatment for 30 min. *N* = 3, ^*^p < 0.05 as compared to untreated.

**Figure 3:**
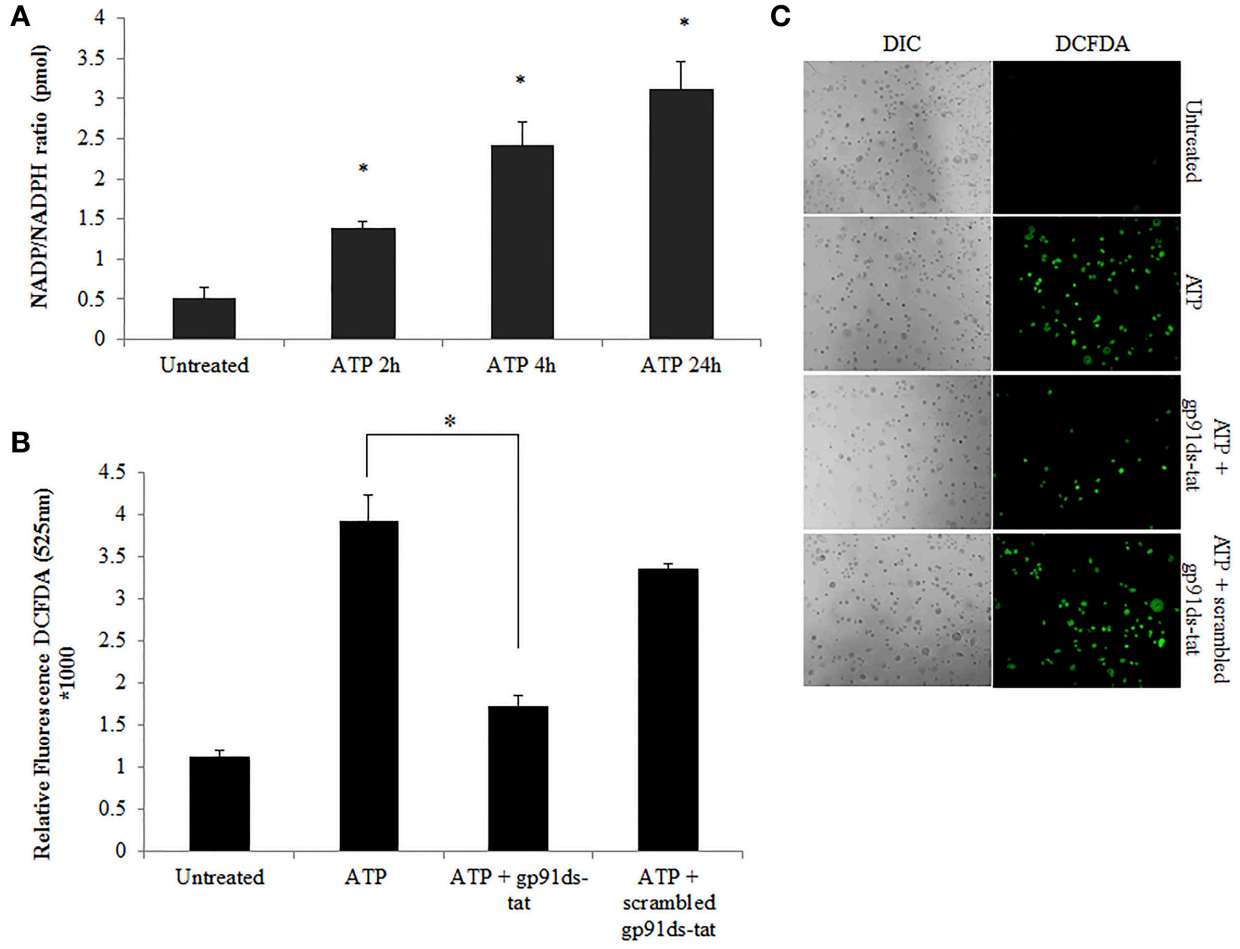
NOX2 is a major contributor to ATP-induced ROS in GECs. **(A)** Measure of NADP/NADPH ratio in ATP (3 mM) treated primary GEC lysates at 2, 4, and 24 h. Increased NADP/NADPH ratio corresponds to more consumed NADPH. *N* = 3, ^*^p < 0.05 as compared to untreated. **(B)** ROS generation in primary GECs after 30 min pre-treatment with ATP (3 mM) and incubation for 2 h with gp91ds-tat peptide (50 μM) (inhibitor of NOX2), or gp91ds-tat scrambled peptide (50 μM) (non-targeting peptide) measured as relative fluorescence using a Biotek H1M monochromatic plate reader at 525 nm. Delivery of gp91ds-tat into primary GECs was confirmed using (50 μM) FAM-labeled peptide (not shown). *N* = 3, ^*^p < 0.05 decrease as compared to ATP. **(C)** Representative micrographs of CM-H_2_DCFDA, ROS probe, live fluorescence imaging combined with Differential Interference Contrast (DIC) microscopy was used for qualitative assessment of the ROS generation with ATP (3 mM) treatment and NOX2 inhibitor peptides shown quantitatively in **(B)**. DIC shows the number of cells for relative comparison. 10x magnification on Leica inverted scope.

**Figure 4:**
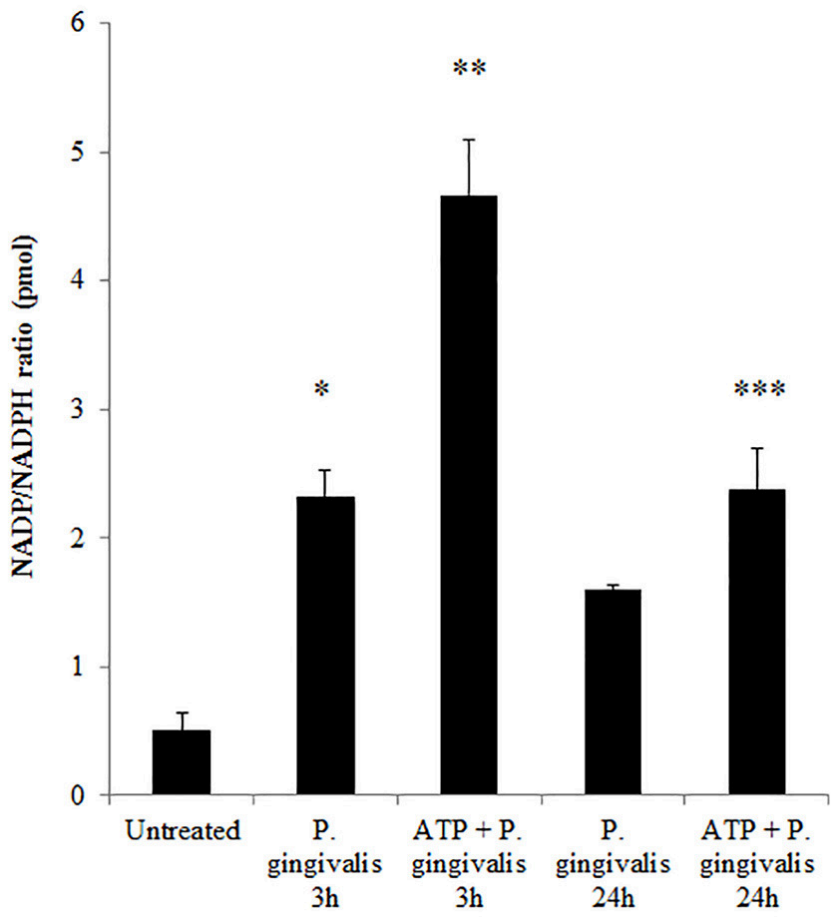
*P. gingivalis* modulates NADPH consumption in GECs. Measure of NADP/NADPH ratio in *P. gingivalis* (MOI100) infected GECs with or without ATP (3 mM) pre-treatment. *N* = 3, ^*^p < 0.05, ^**^p < 0.01, 3 h *P. gingivalis* infection and ATP with 3 h infection as compared to untreated; ^***^p < 0.01 ATP with 24 h infection decrease as compared to ATP with 3 h infection.

Previously, we have shown that *P. gingivalis* infection and eATP induce ROS in primary GECs, largely generated by NADPH oxidases (NOX) inside the cell (Choi et al., 2013). It has been previously determined that eATP treatment of primary GECs results in sustained ROS levels as early as 30 min after treatment (Choi et al., 2013), therefore, we initially examined the mRNA expression of the different NOX isoforms with eATP stimulation to evaluate the NOX isoform important for eATP-induced ROS. The mRNA expression of the seven NOX isoforms in GECS with and without 3 mM ATP treatment revealed differential expression levels whereas NOX 1 and 3 isoforms were not expressed. Among the expressed NOXs, eATP significantly increased NOX2 expression only (Figure 2). We then measured the consumption of NADPH in GECs during ATP treatment using specific detection assays to further demonstrate the activity of NOXs in response to danger signal ATP. The consumption of NADPH steadily increased through 24 h of post-ATP treatment in GECs suggesting increased NOX activity (Figure 3A). However, this ATP-induced NADPH consumption is modulated by *P. gingivalis* infection where the highest increase is at 3 h post-infection that is significantly decreased over 24 h, supporting that *P. gingivalis* modulates NOX activity (Figure 4). The pattern of NADPH consumption is also consistent with the previously published ROS modulation by *P. gingivalis* in the presence of ATP (Choi et al., 2013). We further investigated the specific contribution of NOX2 to eATP-induced ROS using a well-established specific peptide inhibitor against NOX2, gp91ds-tat, which prevents the interaction of the required subunit p47phox to bind to NOX2 (Rey et al., 2001; Csanyi et al., 2011; Park et al., 2011). Treatment of ATP stimulated primary GECs with the NOX2 inhibitor significantly decreased ATP-induced ROS as measured by the fluorescent probe, DCFDA (Figures 3B,C). This supports the specific role of NOX2 in ROS produced by the GECs in response to eATP and infection.

### *P. gingivalis* modulates the activity of NOX2 in primary GECs through the inactivation of critical organizing and regulatory subunits

**Figure 5:**
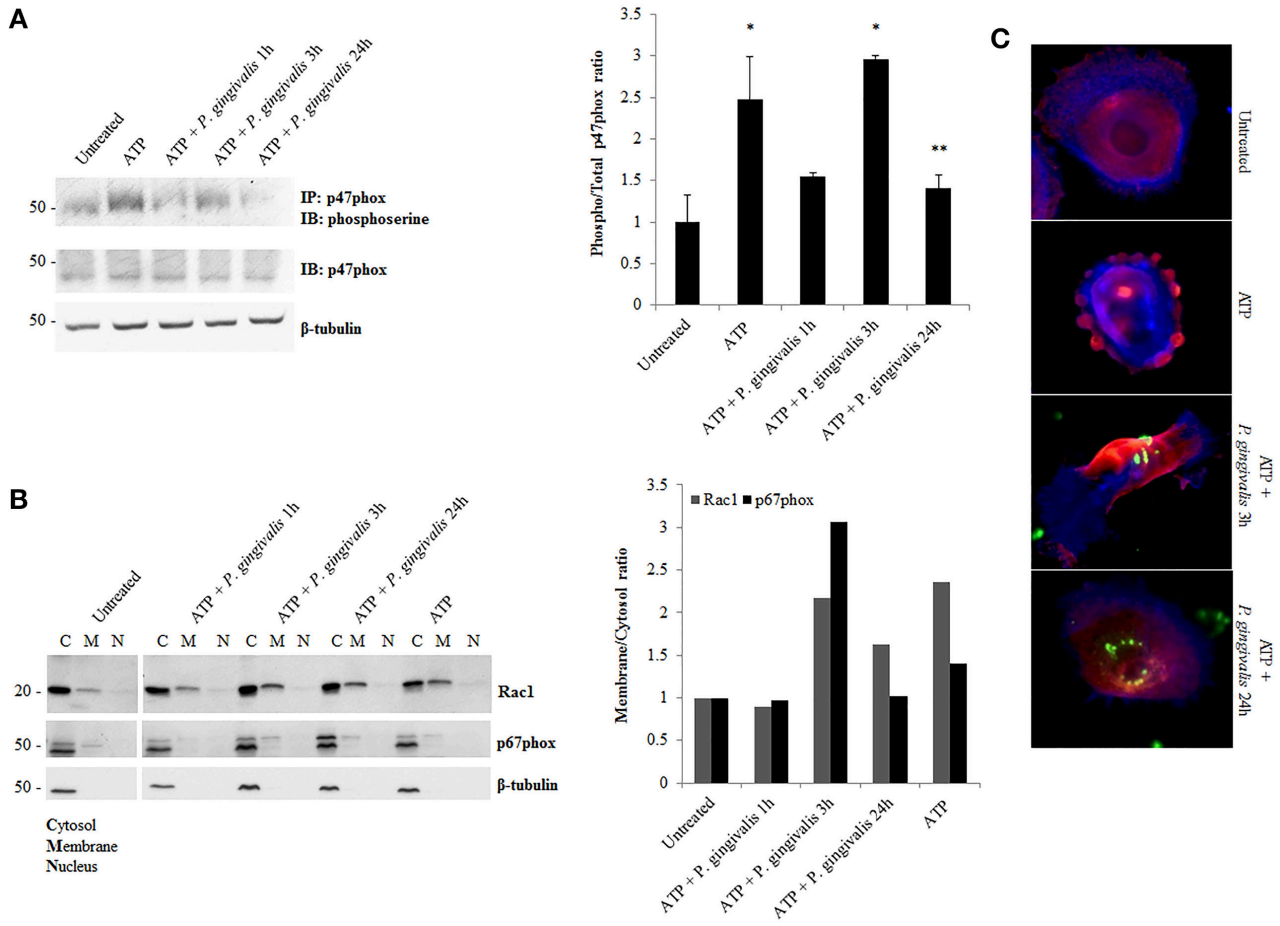
*P. gingivalis* decreases critical NOX2 subunits activation and rearranges subcellular localization. **(A)** Phosphoserine immunoblot of immunoprecipitated p47phox in *P. gingivalis* (MOI100) infected primary GECs with ATP (3 mM) pre-treatment. Quantification of phosphorylated p47phox using NIH Image J Analysis. *N* = 3, ^*^p < 0.05 ATP and ATP with 3 h *P. gingivalis* infection as compared to untreated; ^**^p < 0.05 ATP with 24 h infection decrease as compared to ATP with 3 h infection. **(B)** Subcellular Fractionation of cytosolic, membrane and nuclear fractions in *P. gingivalis* (MOI100) infected and ATP (3 mM) pre-treated cells. Western blot probing for Rac1 and p67phox in primary GEC fractionated lysates. Quantification of Rac1 and p67phox using NIH Image J Analysis. *N* = 3, normalized to beta-tubulin. **(C)** Epifluorescent micrographs of immunofluorescence localization of Rac1 (red) in *P. gingivalis* (green) (MOI100) infected cells. Blue phalloidin staining to outline cellular structure. 20x magnification images on Zeiss Axio Imager.

Although, the expression of NOX2 was increased upon eATP treatment, this may not necessarily suggest increased NOX2 activity. The activation of NOX2 to produce ROS requires the recruitment of specific subunits (Cross and Segal, 2004; Bedard and Krause, 2007). Therefore, we more closely examined the changes in the active forms and localization of these subunits. The critical organizing subunit for the NOX2 complex, p47phox, requires phosphorylation on multiple serine residues to bind to the other subunits in the complex (Hoyal et al., 2003; El-Benna et al., 2009; Meijles et al., 2014). eATP significantly increases the serine phosphorylation of p47phox. *P. gingivalis* modulates the phosphorylation state of p47phox with the most significant increase at 3 h of post-infection which is subsequently reduced over 24 h of post-infection (Figure 5A). This result was consistent with marked increases of ROS previously shown at 3 h of infection which is abrogated over time of infection (Choi et al., 2013). In addition, analysis of the localization of interacting subunits Rac1 and p67phox during ATP treatment and *P. gingivalis* infection reveals similar trends of an increase at 3 h with infection only and decrease at 24 h of *P. gingivalis* infection (Figure 5B). Micrographs of this localization shift in Rac1, provides visual support of the changes which occur following ATP treatment and the infection (Figure 5C). These changes in subunit activation and localization is consistent with the temporal modulation of NOX activity (Figure 4) and eATP-induced ROS kinetics previously described in human primary GECs (Choi et al., 2013).

### *P. gingivalis* reduces ATP-induced hypochlorous acid production in primary GECs thus evading the host biocidal effects

**Figure 6:**
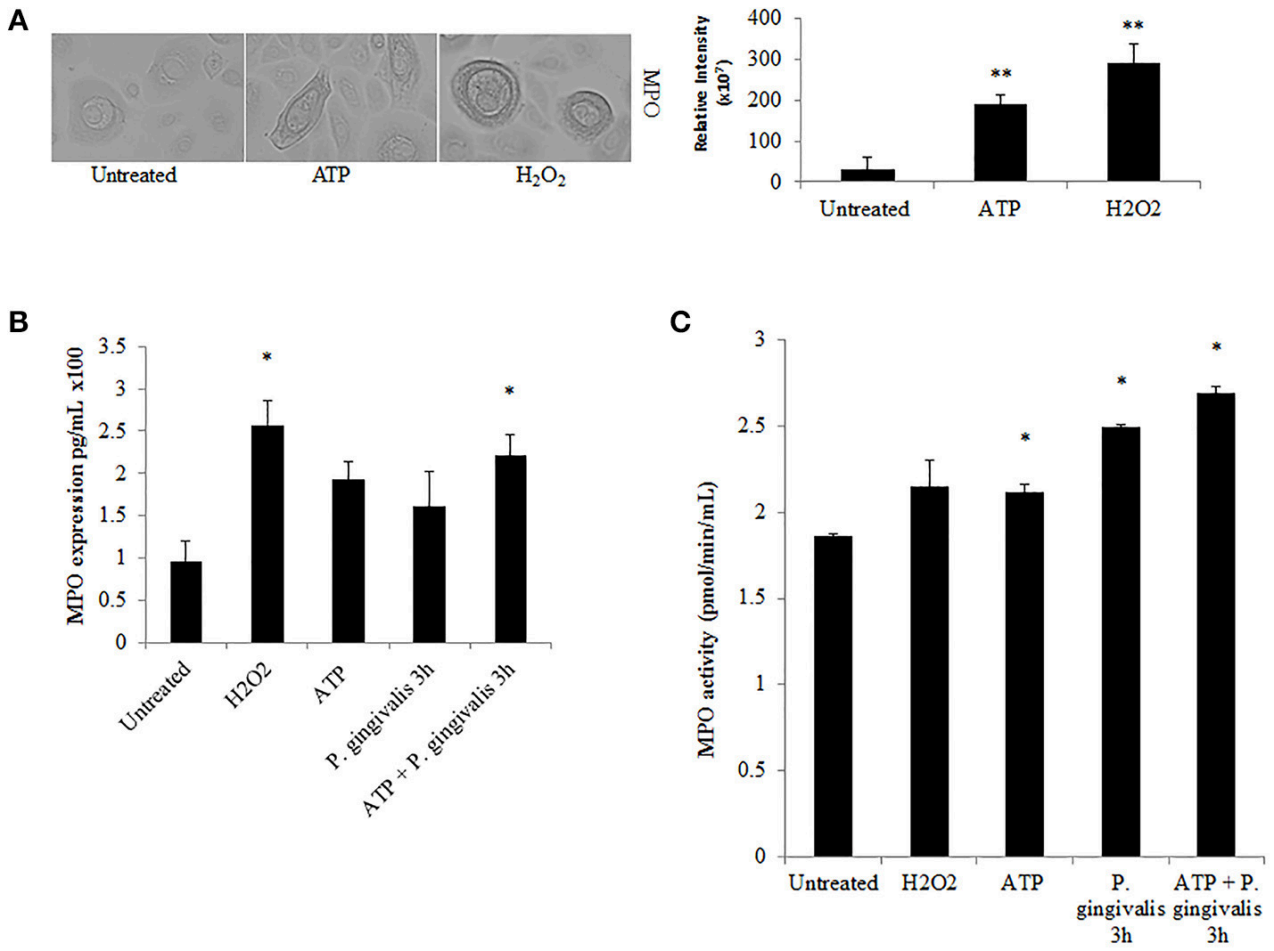
Myeloperoxidase is functionally expressed in primary GECs and activity increases in response to ATP and *P. gingivalis* infection. **(A)** Immunoperoxidase staining of MPO in H_2_O_2_ (100 μM) and ATP (3 mM) cells at 40x magnification performed for visual detection. Image J was used to quantify the relative staining intensity and compared to untreated. *N* = 9, ^**^p < 0.01. **(B)** Quantitative ELISA measurement of MPO expression in primary GECs displayed as pg/mL in H_2_O_2_, ATP pre-treated, and *P. gingivalis* (MOI100) infected cells. *N* = 3, ^*^p < 0.05 as compared to untreated. **(C)** MPO activity assay during ATP pre-treatment and *P. gingivalis* (MOI100) infection in primary GECs calculated based on the standard curve of activity tested empirically (see Methods Section). *N* = 3, ^*^p < 0.05 as compared to untreated.

**Figure 7:**
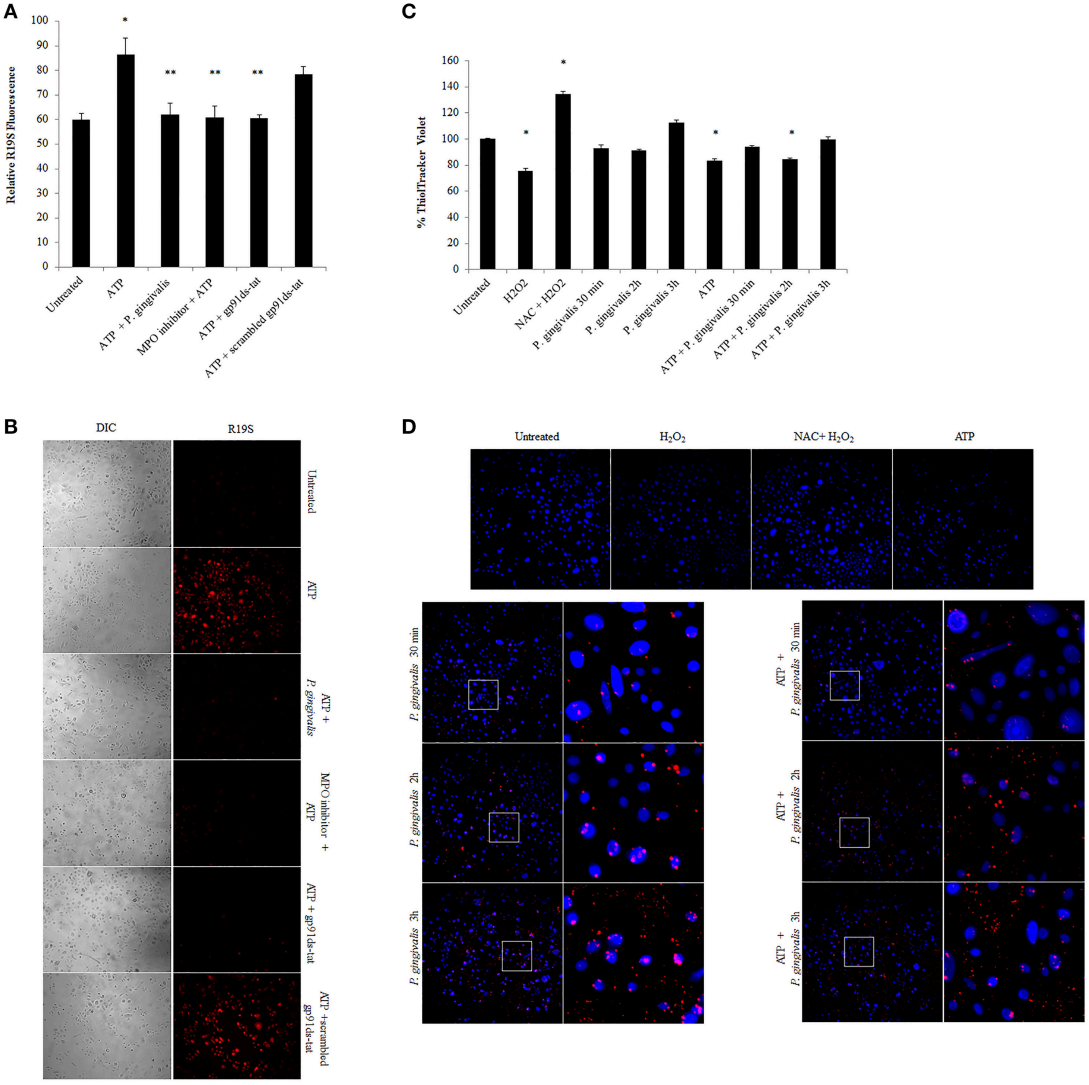
*P. gingivalis* reduces ATP-driven hypochlorous acid production in primary GECs through Glutathione. **(A)** Measure of R19S (10 μM) probe fluorescence, detecting HOCl production, using a Biotek H1M monochromatic plate reader at 545 nm in ATP (3 mM) pre-treated and 2 h *P. gingivalis* (MOI100) infected cells. The MPO inhibitor (1 μM) and gp91ds-tat (50 μM) were added to the cells as described in the Methods Section. *N* = 3, ^*^p < 0.05 ATP compared to untreated; ^**^p < 0.05 inhibitor treatments and *P. gingivalis* infection pre-treated with ATP as compared to ATP only condition. **(B)** Micrographs of live representative images of the R19S probe fluorescence **(A)** in primary GECs. DIC shows the number of cells for relative comparison. 10x Magnification on Leica inverted scope. Concentrations used are ATP (3 mM), scrambled peptide gp91ds-tat (50 μM), MPO inhibitor (1 μM), gp91ds-tat peptide (50 μM). **(C)** Fluorescence quantification of ThiolTracker Violet (20 μM) using Biotek monochromatic plate reader in ATP pre-treated *P. gingivalis* (MOI100) infected cells (30 min; 1, 2, and 3 h) with or without ATP. H_2_O_2_ treatment and N-acetyl cysteine (NAC) were used as positive controls for Thioltracker Violet. Samples were normalized against untreated condition and represented as percent increase or decrease. *N* = 4, ^*^p < 0.05 as compared to untreated. **(D)** Representative micrographs of fixed ThiolTracker Violet (20 μM) (blue fluorescence) was also used to visualize the intracellular reduced GSH levels during ATP pre-stimulation and at early times of *P. gingivalis* (MOI100) infection (30 min; 1, 2, and 3 h) with or without ATP. H_2_O_2_ treatment and N-acetyl cysteine (NAC) were used as positive controls for Thioltracker Violet. *P. gingivalis* infection is confirmed by Alexa fluorophore 594 (red) immunostaining and shown in enlarged micrographs. 10x magnification on Leica inverted scope.

Further downstream of NOX-ROS is the generation of hypochlorous acid (HOCl) by myeloperoxidase (MPO) from H_2_O_2_. Although in neutrophils, MPO is one of the most abundant proteins (Klebanoff et al., 2013), its expression in epithelial cells has not been defined, although is anticipated to be lower. Immunoperoxidase staining in H_2_O_2_ treated primary GECs shows a significant increase in the relative expression of MPO in stressed conditions (Figure 6A). Quantification of MPO expression using an ELISA assay which showed a marked increase in MPO expression in ATP treated cells, and a significant increase with the addition of H_2_O_2_ or pre-ATP treatment and *P. gingivalis* infection (Figure 6B). However, clinical studies reveal that the protein level of MPO may not always provide complete information on the enzymatic activity of MPO which can sometimes vary even if the amount of MPO is similar (Pulli et al., 2013). Therefore, we examined the activity of MPO in primary GECs and show a significant increase in the activity of MPO in ATP treated or *P. gingivalis* infected cells (Figure 6C). Despite this increase in the MPO activity, our data shows that *P. gingivalis* is able to significantly inhibit its downstream product antimicrobial HOCl present at 2 h post-infection (Figures 7A,B). Earlier infection times follow a decreasing trend of HOCl production after 30 min of infection with eATP (Supplementary Figure 2). In addition, the lack of HOCl production in *P. gingivalis* only infected GECs provides evidence for the importance of danger signal ATP signaling in this host innate defense process. Furthermore, the use of the specific inhibitors against MPO (4-aminobenzoic hydrazide) and NOX2 (gp91ds-tat) also significantly decreased HOCl produced in response to ATP. These results suggest that primary GECs induce MPO as a response to infection or stress stimuli such as ATP and that both NOX2 and MPO play major roles in HOCl production against pathogens in primary GECs. However, a well-adapted opportunistic bacterium, *P. gingivalis* appears to have specific molecular strategies to neutralize host ATP-induced HOCl and possibly evade host pathogen clearance.

### *P. gingivalis* increases glutathione enzymes critical for glutathione synthesis in primary GECs

**Figure 8:**
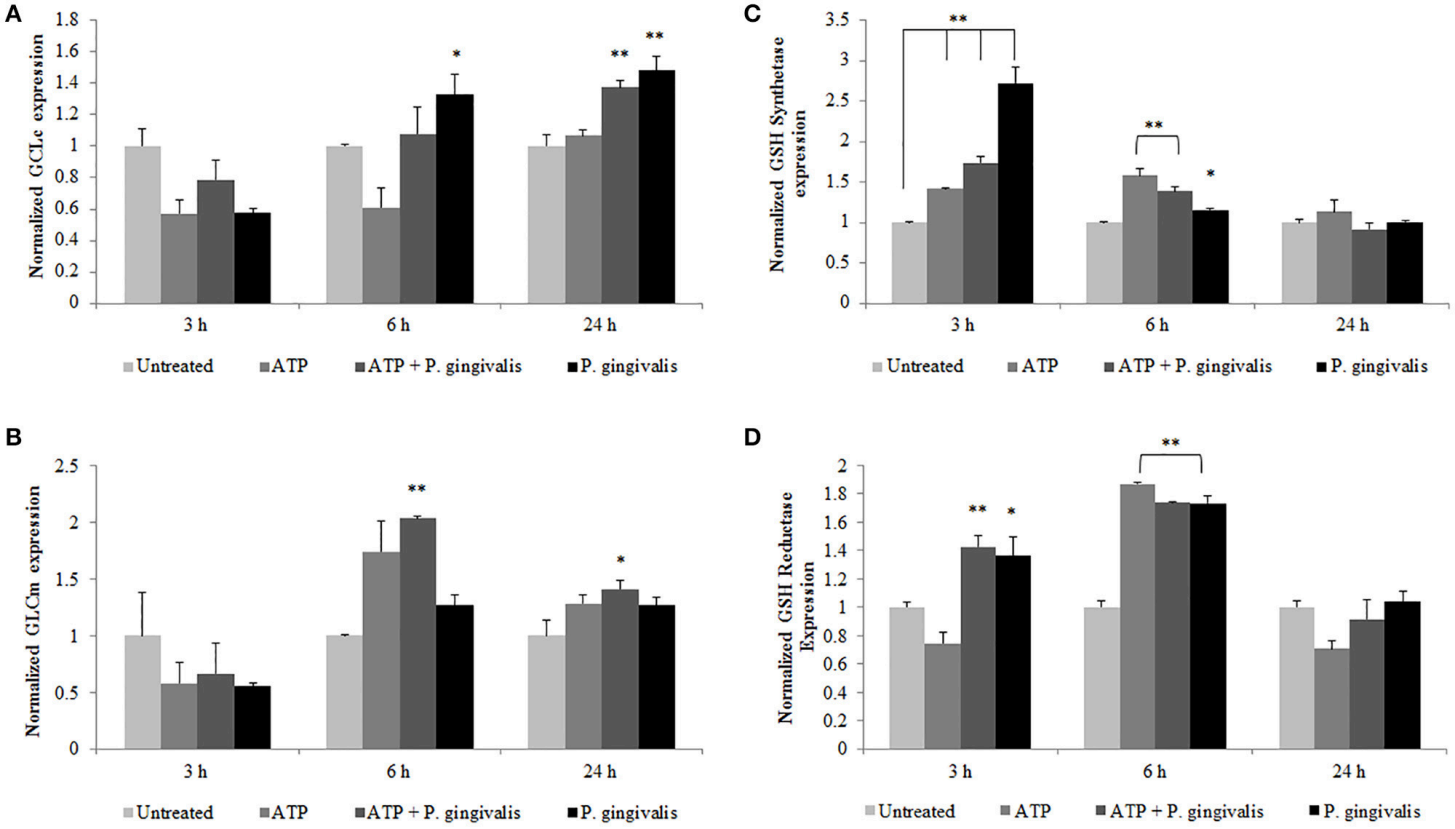
*P. gingivalis* temporally modulates the expression of vital host glutathione renewal and synthesizing enzymes during infection. Primary GECs pre-treated with (3 mM) ATP and infected with *P. gingivalis* (MOI100) for the specified time points were analyzed by quantitative PCR using SYBR Green for mRNA levels of glutathione synthesis enzymes: **(A)** GCLc, **(B)** GCLm, **(C)** glutathione synthetase, and **(D)** glutathione reductase. *N* = 4, ^*^p < 0.05, ^**^p < 0.01 as compared to untreated.

To define a potential molecular mechanism for *P. gingivalis*-mediated reduction in ATP-induced HOCl, we assayed the levels of the critical homeostatic antioxidant glutathione. In its reduced form, glutathione is reactive with HOCl (den Hartog et al., 2002; Venglarik et al., 2003; Gray et al., 2013; Haenen and Bast, 2014). The ThiolTracker Violet dye, which detects the intracellular reduced form of glutathione, revealed a time-dependent oxidative stress response in the presence of ATP in primary GECs, as determined by a decrease in the dye stain (Figures 7C,D). *P. gingivalis*, in early hours of infection in ATP-stimulated GECs also showed a decreasing trend in the reduced glutathione levels with a significant decline at 2 h of infection which suggests that the antioxidant is scavenging ROS and/or HOCl (Figures 7C,D). Furthermore, depletion of glutathione synthesis using buthionine sulfoximine (BSO; Markovic et al., 2009; Rocha et al., 2014) abrogated *P. gingivalis'* ability to lessen ATP-induced HOCl which supports a role for glutathione in scavenging the HOCl during early *P. gingivalis* infection (Supplementary Figure 3). We therefore examined the mRNA expression of key enzymes for glutathione synthesis (Lu, 2013; GCLc and GCLm, glutathione synthetase, and glutathione reductase). The expression levels of GCLc and GCLm, the subunits of the glutamate cysteine ligase (GCL), are significantly increased by *P. gingivalis* infection at 6 and 24 h of infection even in the presence of ATP. GCL is a critical rate-limiting step in glutathione synthesis (Lu, 2013), therefore the increase of expression suggests that *P. gingivalis* is inducing more glutathione production. Moreover, the second step in glutathione synthesis requires glutathione synthetase which is significantly increased by *P. gingivalis* at 3 h post-infection and reduced at 24 h. Glutathione reductase, which reduces oxidized glutathione, follows a similar pattern and is significantly increased at 3 and 6 h post-infection, but returns to normal levels at 24 h (Figure 8). It is important to note that at 3 h of *P. gingivalis* infection with and without ATP treatment there is a notable increase in glutathione levels at 3 h of infection as compared to the reduced amount at 2 h of infection (Figures 7C,D). The increased expression of glutathione synthetase and reductase at this same time during *P. gingivalis* infection suggests *P. gingivalis* can induce host glutathione production as a combative antioxidant response to ATP-induced cellular HOCl. Furthermore, we have previously described (Choi et al., 2013) and also reconfirm using ThiolTracker that 24 h *P. gingivalis* infection had comparable levels of reduced glutathione detected by ThiolTracker to untreated with and without ATP treatment (not shown). Taken together, these findings suggest that *P. gingivalis* can control the cellular glutathione synthesis pathway and utilize host glutathione to scavenge cellular HOCl. By further increasing the key synthesizing enzymes necessary, *P. gingivalis* can restore the reduced glutathione levels in primary GECs and promote an intracellular environment likely conducive for prolonged bacterial survival and persistence.

## Discussion

The physical and biological barrier created by the mucosal epithelium most often involves highly sophisticated interactions with invading bacteria in the human body (Dale, 2002, 2003). Similarly, oral bacteria that colonize in oral mucosa have developed elaborately specific strategies to subvert the immune system (Colombo et al., 2006, 2007). The Gram (−) anaerobe, *P. gingivalis*, a key member of the subgingival microbiome, can invade, replicate, and successfully survive in GECs and subsequently spread to the surrounding cells without alerting the immune system (Lamont and Yilmaz, 2002; Yilmaz et al., 2006; Hung et al., 2013). These cellular events involve the manipulation of multiple molecular pathways involved in inhibition of host cell death and oxidative stress including the P2X_7_-NADPH oxidase interactome (Yilmaz et al., 2004, 2008; Yao et al., 2010; Choi et al., 2013).

Metabolic profiling in recent clinical studies documents the significance of purinergic signaling in the human oral cavity revealing high levels of purines during inflammation in the gingival crevicular fluid and saliva (Jahngen et al., 1984; Barnes et al., 2009; Lim and Mitchell, 2012). Furthermore, numerous studies indicate the importance of P2X_7_ ligation with danger signal, eATP, released from infected or stressed cells, in mucosal immunity (Gordon, 1986; Surprenant et al., 1996; Schwiebert and Zsembery, 2003; Moore and MacKenzie, 2009; Trautmann, 2009; Hung et al., 2013). The eATP/P2X_7_-mediated signaling is largely linked to high levels of cellular ROS important for the facilitation of pathogen clearance and activation of specific immune defense mediators in specialized immune cells (Yilmaz, 2008; Trautmann, 2009; Yilmaz et al., 2010; Miller et al., 2011; Spooner and Yilmaz, 2011; Choi et al., 2013; Di Virgilio and Vuerich, 2015; Almeida-da-Silva et al., 2016). More recently, these pathways have been more clearly defined in the gingival epithelium and accumulated evidence in the last decade has indicated potentially an important role for eATP-mediated cellular ROS in the pathogenesis of periodontal disease (Battino et al., 1999; Waddington et al., 2000; Chapple and Matthews, 2007).

**Figure 9:**
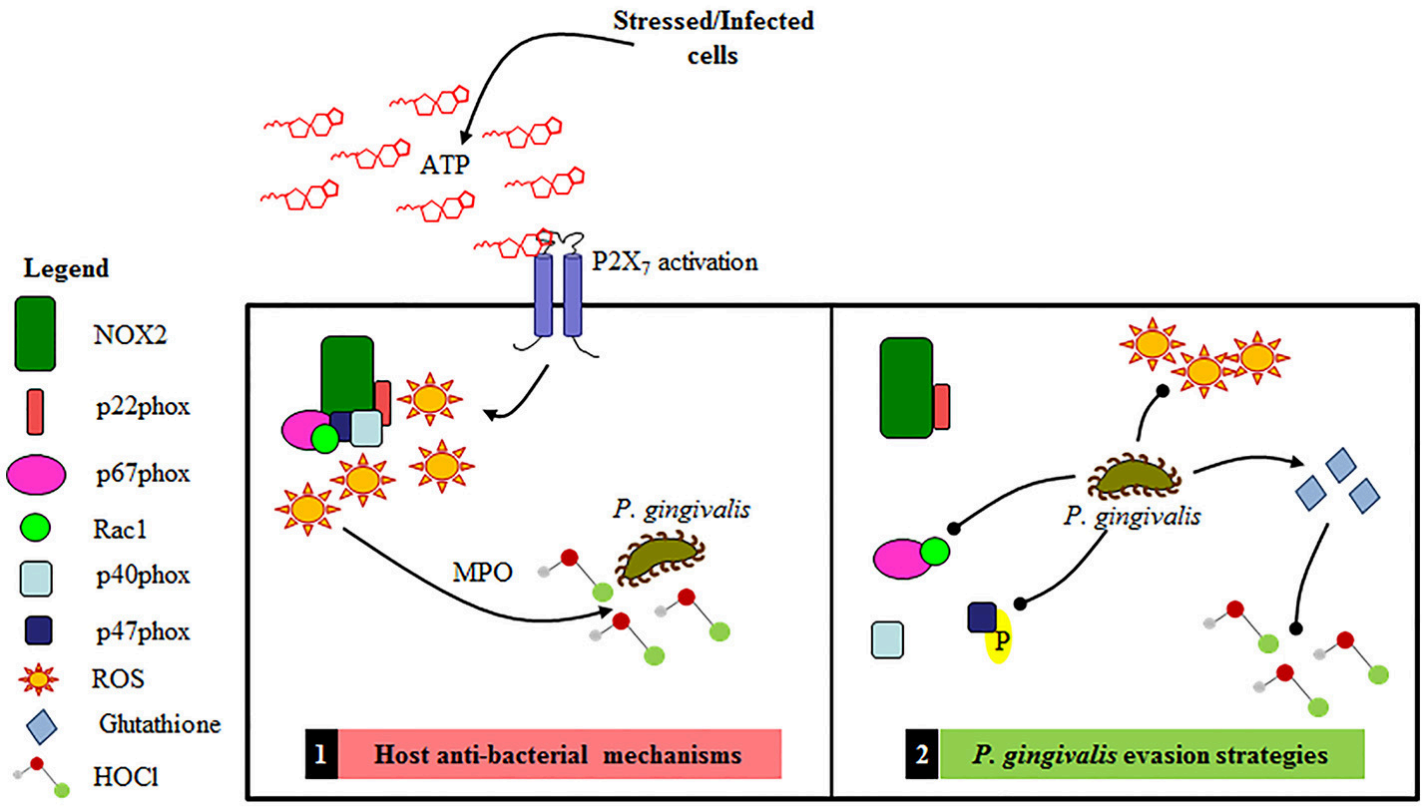
Schematic representation of the proposed Gingival Epithelial Cell (GEC) anti-bacterial response and *P. gingivalis'* specific evasions strategies of NOX2 antibacterial machinery. 1. Primary GEC host anti-bacterial mechanisms: NOX2-ROS is produced in response to eATP signaling leading to the production of HOCl and bacterial elimination. 2. *P. gingivalis* evasion strategies: *P. gingivalis* temporally blocks the formation of the NOX2 complex by affecting activation of critical cytosolic subunits and also diminishes ATP mediated HOCl production via the scavenging antioxidant glutathione.

We previously reported a significant modulation in cellular ROS levels as a result of the P2X_7_/NADPH oxidase (NOX) interactome and mitochondrial crosstalk activated by the release of ATP upon *P. gingivalis* infection in the GECs (Choi et al., 2013). However, the specific NOX critical for generation of anti-bacterial ROS, defined here as ‘ROS promoting activation of host bacterial killing mechanism,’ was not explored. The NOX2 isoform of the NOX family is well-characterized for bacterial clearance in professional phagocytes and is at least partially dependent on P2X_7_ activation by eATP (Cross and Segal, 2004; Lambeth, 2004; Moore and MacKenzie, 2009; Choi et al., 2013). Moreover, there is some evidence in literature which indicates other bacteria that are able to attenuate NOX activation in neutrophils for persistence (e.g., *Francisella tularensis* and *Anaplasma phagocytophilum*; Carlyon et al., 2004; McCaffrey and Allen, 2006; Sun et al., 2013). Nevertheless, there are currently no studies showing a functional role for NOX2 and associated bacterial clearance mechanisms such as myeloperoxidase activity in mucosal epithelial cells or in the oral cavity. Our study demonstrates for the first time eATP-stimulated NOX2 presence and activity in human primary GECs and subsequent generation of biocidal products such as hypochlorous acid (HOCl) summarized in Figure 9. Furthermore, we demonstrate that *P. gingivalis* infection successfully rearranges and shifts the presence of active or inactive components (p47phox, p67phox, and Rac1) of the NOX2 complex at various time points of infection. The modulation of these critical NOX2 subunits is consistent with the high and low levels of ROS modulated by *P. gingivalis* infection as previously reported (Choi et al., 2013). It is perhaps possible that the ROS production can aid *P. gingivalis* for other aspects of its intracellular life cycle as a secondary signaling molecule rather than a noxious agent similar to other chronic bacteria like *Chlamydia trachomatosis* (Abdul-Sater et al., 2010). It is tempting to suggest that *P. gingivalis* as a well-adapted human pathogen, could sense cues in an orchestrated way that it can distinguish ROS initially as an alarming signal or not. It is important to note, however, that early spikes of cellular ROS generation in response to infection are more commonly associated with bacterial clearance (Nauseef, 2007; Spooner and Yilmaz, 2011; Paiva and Bozza, 2014). Furthermore, H_2_O_2_ specifically is catalyzed by myeloperoxidase (MPO) to produce hypochlorous acid (HOCl) in the presence of bacterial infection as described in neutrophils (Hampton et al., 1998; Nauseef, 2014). We demonstrated the increased expression of MPO in response to stress stimuli in GECs, which is consistent with a recent clinical study that detected high-intensity MPO staining in the epithelial layers of human gingiva during periodontal inflammation (Kuzenko et al., 2015). Intriguingly, GECs significantly increase MPO activity in response to exogenous ATP treatment and *P. gingivalis* infection whereas the data also demonstrate a significant decrease in the downstream MPO reaction product, HOCl, in the presence of infection and ATP. The antioxidant glutathione has recently been investigated for its ability to effectively scavenge HOCl before damage is inflicted (Pullar et al., 2001; den Hartog et al., 2002; Venglarik et al., 2003; Haenen and Bast, 2014). Previously, we have shown *P. gingivalis* infection increases host cell glutathione in the presence of eATP and in primary human GECs as a means of intracellular survival (Choi et al., 2013), which suggests this could be a likely mechanism for the decrease in eATP-driven HOCl observed during early infection. The ThiolTracker Violet data showed a decrease of reduced glutathione levels at early times of *P. gingivalis* infection which coincides with the observed reduced HOCl levels. This suggests that glutathione is being used to combat oxidative stress during early *P. gingivalis* infection and is likely also scavenging the HOCl. This is a likely assumption (1) because HOCl is 100-fold faster in reactivity with glutathione and other sulfur-containing compounds than it is with other components inside the cell (Gray et al., 2013; Haenen and Bast, 2014) and (2) because glutathione is an abundant, non-protein thiol critically important for redox signaling (Lu, 2013).

In further support of this proposed mechanism of HOCl scavenging by glutathione, we show depletion of glutathione abates *P. gingivalis*' ability to reduce HOCl levels. In addition, mRNA expression of vital enzymatic machinery important in the biosynthesis and recycling of intracellular glutathione points to a strategic and timed manipulation of the host antioxidant system by *P. gingivalis* in order to maintain the balance of the cellular redox environment in GECs and thereby promote a pro-survival phenotype. More specifically, *P. gingivalis* increases the mRNA expression of (1) glutathione synthetase, which catalyzes the second step in the glutathione synthesis pathway, (2) glutathione reductase, which converts the oxidized form of glutathione back into the reduced form, and (3) the two subunits (GCLc and GCLm) which form a holoenzyme called GCL and catalyzes the rate-limiting step in the glutathione synthesis pathway (Lu, 2013). It is important to note that the glutathione synthetase and reductase are increased at earlier times of infection (~3–6 h) when an increase in the reduced glutathione levels are observed again with *P. gingivalis* and ATP. The GCL however is increased at 24 h of infection demonstrating that *P. gingivalis* induces different steps of the glutathione synthesis pathway over the course of infection, thus promoting continued glutathione synthesis to contest eATP and infection triggered host oxidative stress. A well-coordinated modulation of the host redox pathways by *P. gingivalis* perhaps enables the microorganism to shift the cellular environment to more favorable conditions for continued survival and persistence in the oral cavity. Although this particular study is not geared to explore the bacterial effector(s) which may neutralize HOCl, it is tempting to consider that *P. gingivalis* may additionally express a HOCl scavenger enzyme or enzyme system like some aerobic bacteria produce (Chesney et al., 1996; Gray et al., 2013).

Finally, while our study shows for the first time NOX isoforms expressed in human GECs, it demonstrates NOX2 to be the major ROS contributor in the presence of eATP and *P. gingivalis* infection and presents potentially unique strategies used by *P. gingivalis* to perhaps evade HOCl-mediated pathogen clearance. While further studies are warranted to determine the exact mechanisms and biological importance of these molecular interactions for the bacterial persistence; overall, this novel study lays the groundwork for advancing our current understanding of host-pathogen interactions in the epithelial tissues which may lead to specific therapeutic strategies against successful opportunistic bacteria such as *P. gingivalis*. Studies in the future investigating NOX2 and associated anti-bacterial defense mechanisms may also provide useful strategies for targeting microbial factors involved in human chronic diseases in mucosa.

## Author contributions

Conceived and designed the experiments and wrote the paper: JR and ÖY. Performed the experiments: JR, CC and JD. Aided with Experiments: KA and JL. Analyzed the data: JR, KA, JL, GD, CC, JD and ÖY. Contributed reagents/materials/analysis tools: ÖY and GD.

### Conflict of interest statement

The authors declare that the research was conducted in the absence of any commercial or financial relationships that could be construed as a potential conflict of interest.
