# Supplementary material for: Opportunistic Pathogen Porphyromonas gingivalis Modulates Danger Signal ATP-Mediated Antibacterial NOX2 Pathways in Primary Epithelial Cells
Source: Front Cell Infect Microbiol. 2017 Jul 4;7:291. doi: 10.3389/fcimb.2017.00291 (PMC5495830; doi:10.3389/fcimb.2017.00291)
Supplement: Supplementary file 1 [file Presentation1.pdf]

Supplementary Figures

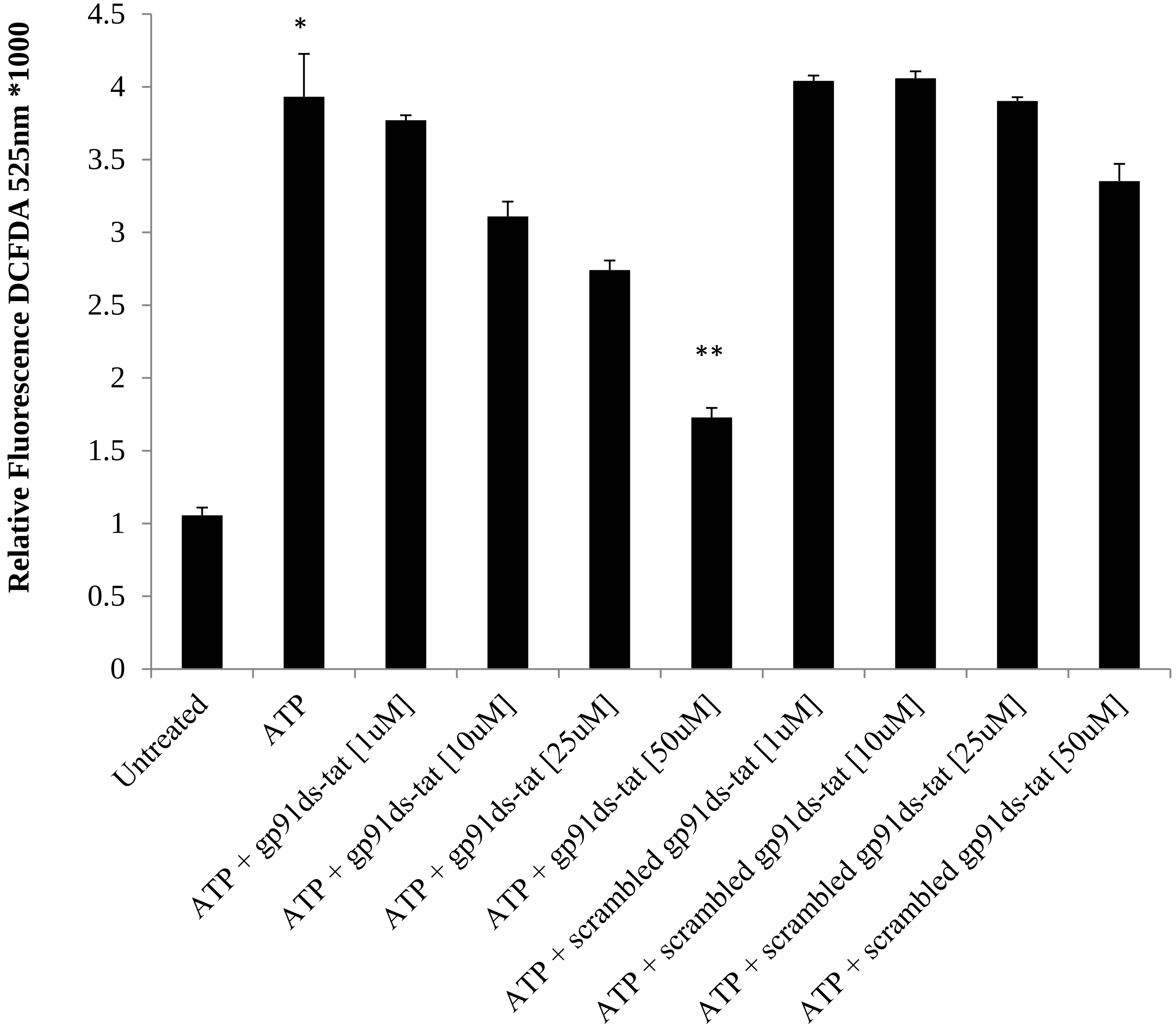

Supplementary Figure 1. Dose-dependent decrease in ATP-induced ROS production in response to gp91ds-tat NOX2-specific peptide inhibitor in primary GECs. ATP significantly increases ROS, which is significantly decreased in gp91ds-tat [50µM] treated cells as compared to ATP-treated *p<0.05 as compared to untreated; **p<0.05 as compared to ATP treated.

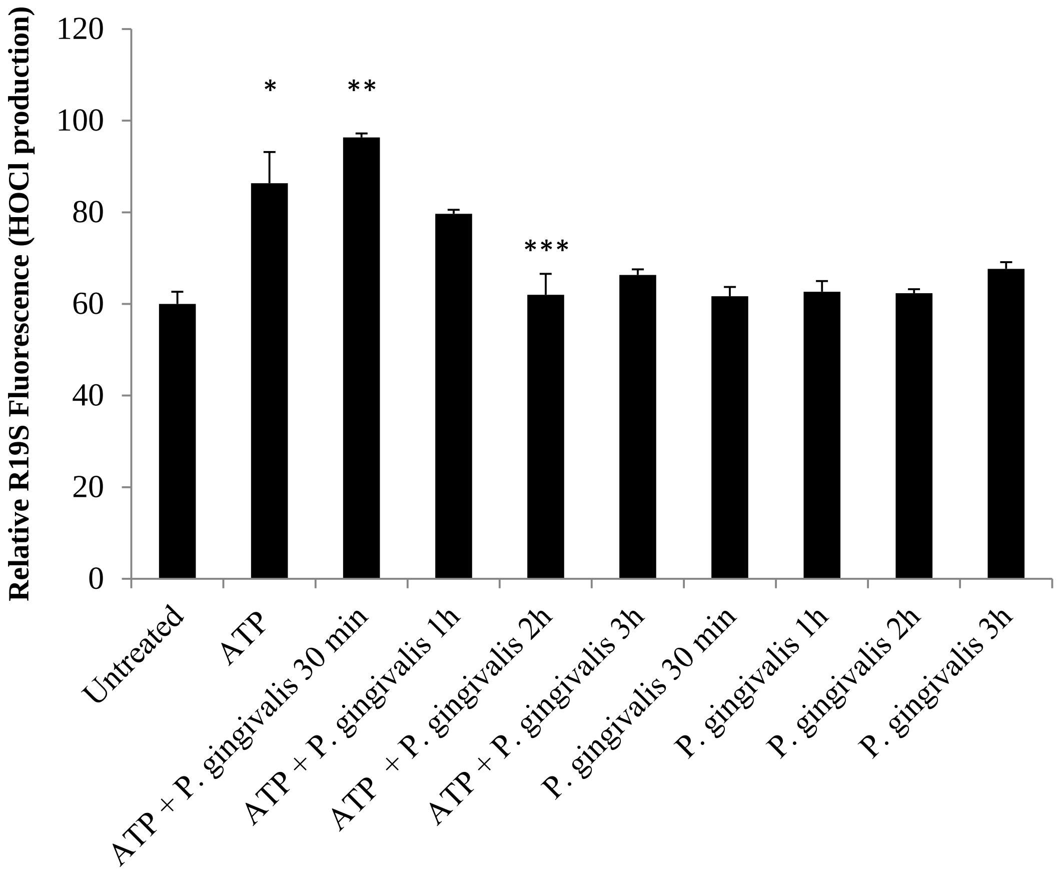

Supplementary Figure 2. Hypochlorous acid (HOCl) production in primary GECs at early times of *P. gingivalis* infection with and without ATP treatment. Measure of R19S probe fluorescence [10µM], detecting HOCl production, using a Biotek H1M monochromatic plate reader at 545 nm in ATP [3mM] treated and *P. gingivalis* infected cells (MOI100). ATP significantly increases HOCl production alone and in early time of infection (30min) which is significantly decreased after 2 hours of infection. N=3, *p<0.05, **p<0.01 as compared to untreated; ***p<0.05 as compared to ATP treated.

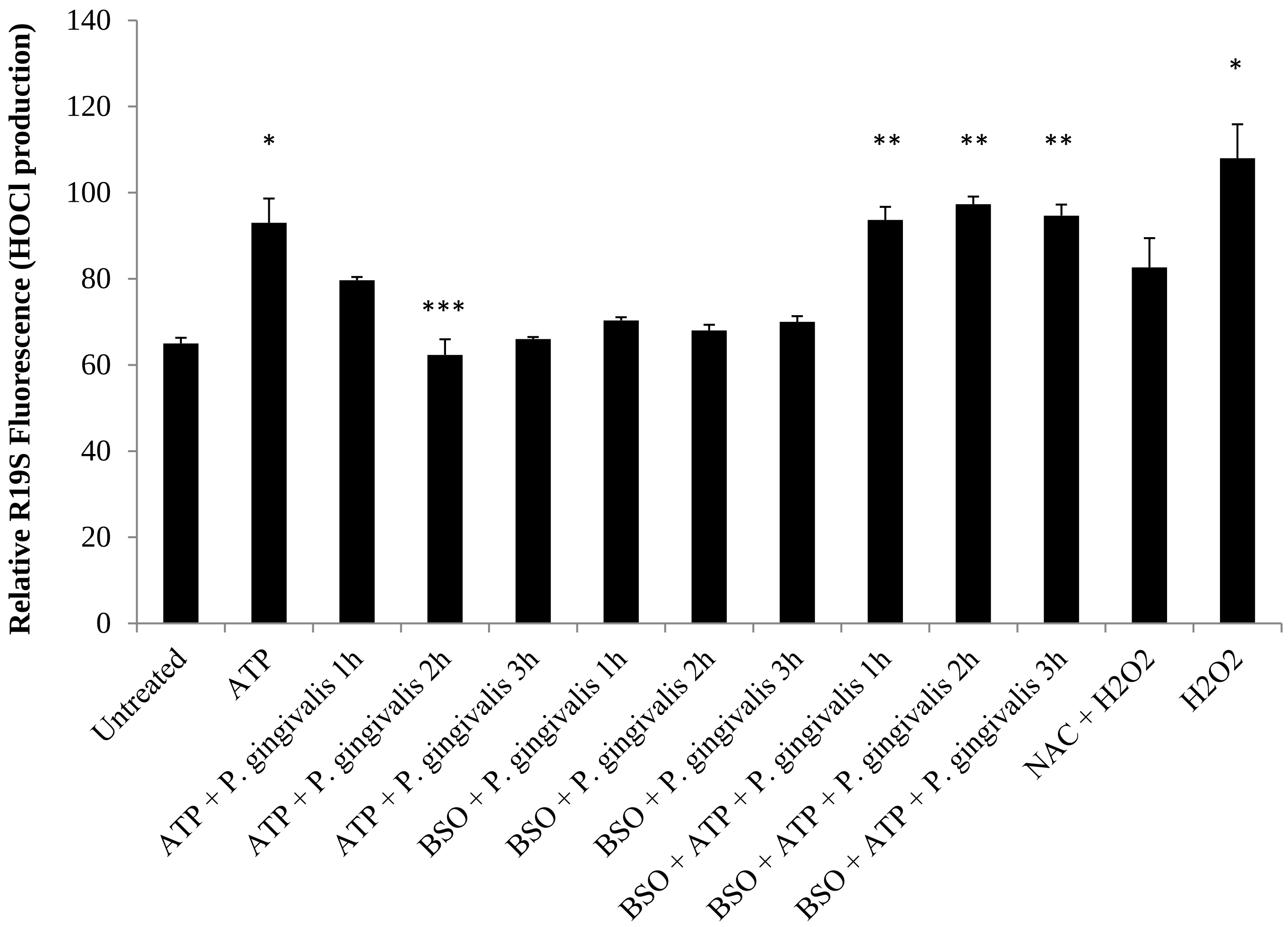

Supplementary Figure 3. Glutathione depletion diminishes the ability of *P. gingivalis* to decrease ATP-induced HOCL production in primary GECs. Buthionine sulfoximine (BSO) [100µM] was added 24 hours prior to *P. gingivalis* infection (MOI100) and/or [3mM] ATP treatment. N-acetyl cysteine (NAC) [50µM] reduced H_2O_2 mediated HOCl production as a positive control for glutathione synthesis. HOCl production was measured using the R19S fluorescent probe on a Biotek H1M monochromatic plate reader at 545 nm. N=3, *p<0.05, **p<0.01 as compared to untreated; ***p<0.05 as compared to ATP treated.
